# Time-of-Day Variation in SARS-CoV-2 RNA Levels during the Second Wave of COVID-19

**DOI:** 10.3390/v14081728

**Published:** 2022-08-05

**Authors:** Xiaodong Zhuang, Wei Wang, Helene Borrmann, Peter Balfe, Philippa C. Matthews, David W. Eyre, Elizabeth B. Klerman, Jane A. McKeating

**Affiliations:** 1 Nuffield Department of Medicine, University of Oxford, Oxford OX3 7FZ, UK; 2 Division of Sleep and Circadian Disorders, Brigham and Women’s Hospital, Division of Sleep Medicine, Harvard Medical School, Boston, MA 02115, USA; 3 The Francis Crick Institute, London NW1 1AT, UK; 4 Division of Infection and Immunity, University College London, London WC1E 6BT, UK; 5 Department of Infection, University College London, Hospital NHS Foundation Trust, London WC1E 6BT, UK; 6 Big Data Institute, Nuffield Department of Population Health, University of Oxford, Oxford OX3 7LF, UK; 7 Division of Sleep Medicine, Harvard Medical School, Boston, MA 02115, USA; 8 Department of Neurology, Massachusetts General Hospital, Boston, MA 02114, USA; 9 Chinese Academy of Medical Sciences Oxford Institute, University of Oxford, Oxford OX3 7FZ, UK

**Keywords:** circadian, SARS-CoV-2, RNA, diurnal, COVID-19

## Abstract

Circadian rhythms influence and coordinate an organism’s response to its environment and to invading pathogens. We studied the diurnal variation in severe acute respiratory syndrome coronavirus 2 (SARS-CoV-2) RNA in nasal/throat swabs collected in late 2020 to spring 2021 in a population immunologically naïve to SARS-CoV-2 and prior to widespread vaccination. SARS-CoV-2 diagnostic PCR data from 1698 participants showed a significantly higher viral load in samples obtained in the afternoon, in males, and in hospitalised patients when linear mixed modelling was applied. This study illustrates the importance of recording sample collection times when measuring viral replication parameters in clinical and research studies.

## 1. Introduction

COVID-19 caused by SARS-CoV-2 is one of the greatest health challenges we have faced in the 21st century. The concerted effort of academia, industry, government and regulatory bodies has resulted in effective vaccines and drug discovery programmes that limit SARS-CoV-2 transmission and disease severity [1]. At the time of writing, more than 5 million people have died, and the pandemic remains a global challenge. Understanding host pathways and associated factors that define susceptibility to SARS-CoV-2 infection and disease severity will inform future clinical management and public health measures to control this disease.

Circadian rhythms are endogenous daily oscillations that influence and coordinate an organism’s response to its environment. Many aspects of host immunity are regulated by the circadian clock and these immune rhythms are likely to have evolved to defend against diurnal peaks of pathogen encounters (reviewed in [2]). A recent report demonstrated significant time-of-day variation in multiple immune parameters, including lymphocyte and neutrophil counts in >300,000 participants in the UK Biobank, highlighting the rhythmicity in innate and adaptive immune responses [3]. In models of viral or bacterial infection, genetic disruption of the circadian clock increase disease severity [4,5,6,7,8,9,10,11]. Lung diseases frequently show time-of-day variation in respiratory function and severity of symptoms [12], with the key circadian component, BMAL1, regulating inflammation [13]. Influenza A virus infection of mice lacking BMAL1 showed a higher viral burden in the lung [14] and elevated inflammatory responses [4,11]. There is an emerging picture of time-of-day dependency of virus replication (reviewed in [15]), suggesting that circadian regulation of infection is ubiquitous.

We recently identified a role for BMAL1 in regulating SARS-CoV-2 infection in vitro [16], suggesting that virus replication may vary during the day, and this could influence transmission. To explore the relevance of this observation, we performed a retrospective study to assess the relationship between SARS-CoV-2 RNA levels in nasal/throat swabs and time of sample collection in a cohort of 1698 adults tested by the Oxford University Hospitals, UK, from late 2020 to Spring 2021. This period covered the second wave of SARS-CoV-2 transmission in the UK involving the Alpha variant and was prior to widespread vaccination, providing an opportunity to study the daily variation in SARS-CoV-2 RNA levels in an immunologically naïve population.

## 2. Methods

### 2.1. Sample Collection

Samples were obtained from adults on hospital wards or admission units, classified as in-patients, or from out-patient centres. As health care workers were prioritized for SARS-CoV-2 vaccination in the UK from late December 2020, they were excluded from our analysis. We obtained anonymised SARS-CoV-2 PCR data from combined nasal/throat swabs collected from Nov 2020 to May 2021 from the Infections in Oxfordshire Research Database with Research Ethics Committee approvals (19/SC/0403 and ECC5-017(A)/2009). The following information was available: age, sex, time of sample request and time of receipt in diagnostic laboratory.

### 2.2. SARS-CoV-2 RNA Quantification

Viral RNA was measured using the Thermo Fisher TaqPath COVID-19 RT-PCR Kit that measures ORF1ab, Spike (S) and Nucleocapsid (N) gene transcripts. As an internal control for the quality of RNA isolation clinical samples were supplemented with MS2 phage RNA prior to extraction. S gene amplification will fail when the infecting variant has genetic mutations or deletions in S, and these samples are defined as S Gene Target Failures (SGTF). Sequencing of viruses showed that the Victoria (VIC) strain was circulating at this time and the SGTF samples represented infection with the Alpha variant (Δ69/70 Spike) [13]. Viral loads (VLs) were estimated from the Ct values using standard curves for each amplicon, as previously reported [12,13]. The interval between sample request and laboratory receipt times allowed us to assess the effect of transport or storage delays on the VL estimates. The VL in samples with intervals of 0–3 or 3–6 h showed a median Log_10_ VL of 4.2 and 3.9, respectively; however, samples with an interval >6 h had a reduced Log_10_ VL of 3.7. To reduce variance in VL and to increase confidence in sample time for statistical evaluation, we selected samples with a <6 h interval and used the sample request time for all analysis.

### 2.3. Statistical Analysis

We selected to use a linear mixed-effects model that can account for multiple factors known to influence VL measurements. VLs were log10-transformed and the sample request time (6:00–11:59 a.m. vs. 12:00–17:59 p.m.), age groups (18–39, 40–59, 60–79 and 80–104 years), sex (male vs. female), location (in-patients vs. out-patients) and virus strain (VIC vs. Alpha), together with their interactions, were included in the model as fixed effects. Participants were treated as random effects to account for inter- and intra-individual variability. Additional analyses were performed where the sample request time was classified into three intervals (morning 6:00–9:59 a.m., mid-day 10:00 a.m.–13:59 p.m. and afternoon 14:00–17:59 p.m.) or age was considered as a continuous variable. To assess the non-linear effects of age on VL, a B-spline fit of participant age was modelled [17], with residual plots used to check model assumptions and goodness of fit. Statistical analyses were performed using SAS version 9.4 (SAS Institute, Cary, NC, USA) with the significance level set at α = 0.05. All tests were two-sided.

## 3. Results

**Figure 1:**
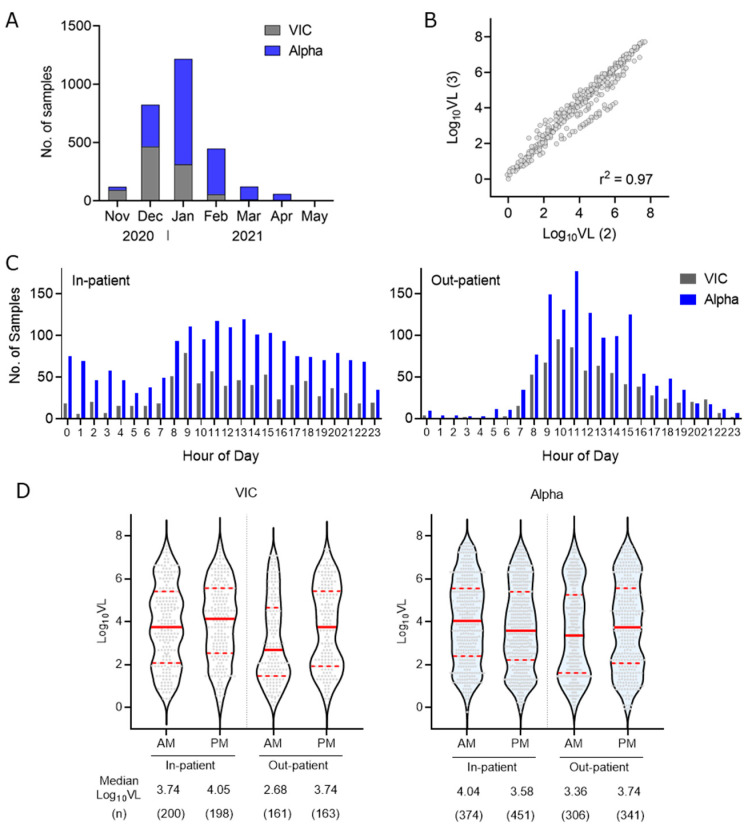
(**A**) Monthly COVID-19 PCR samples detected in Oxford UK as Victoria (VIC, grey) or Alpha (blue) strain between Nov 2020 and May 2021. (**B**) Viral Load (VL) estimates for the VIC samples derived from either 2 (ORF1, N; *x*-axis) or 3 (ORF1, N and S; *y*-axis). Ct values agreed closely (r^2^ = 0.97). (**C**) Sample request times from in-patient and out-patient groups listed by hour of the day and by VIC or Alpha strain. (**D**) Violin plots of Log_10_ VL of the infecting virus strain (VIC/Alpha), partitioned by in- and out-patient groups (location) and by sample time (a.m., 06:00–11:59; p.m., 12:00–17:59). Median Log_10_ VL is depicted with a solid red bar and the interquartile range with hashed red lines.

The SARS-CoV-2 VIC and Alpha strains were co-circulating in Nov–Dec 2020, whereas by Apr–May 2021 the Alpha strain accounted for >90% of infections (Figure 1A). To compare VIC and Alpha RNA levels, we estimated the VL for VIC using either two (ORF1 and N) or three (ORF1, N and S) amplicons and observed an excellent agreement (r^2^ = 0.97) (Figure 1B); we therefore used the ORF1 and N Ct values to estimate VL for all samples. In total, 85% of sample request times occurred during the working day (06:00–18:00) (Figure 1C), leading us to partition the data into morning (a.m.: 6:00–11:59) and afternoon (p.m.: 12:00–17:59). Participants were classified into four age groups (18–39, 40–59, 60–79 and 80–104 years) (Table 1).

Several factors in this dataset suggest that simple univariate analyses are not appropriate: (i) in-patients (n = 1223) had a higher VL than out-patients (n = 969) (mean Log_10_ VL 3.87 vs. 3.61, *p* < 0.003) and (ii) in-patients were older (median of 66 vs. 54 years, *p* < 0.0001) and included a higher proportion of males (52% vs. 48%, *p* = 0.099). We therefore selected a linear mixed-effects modelling approach to assess the effect of sample request time, age, sex, in-patient vs. out-patient and virus strain (VIC vs. Alpha) as fixed effects on VL. Our analysis showed the VL associated with: (i) sample request time, with an estimated log_10_ VL of 3.56 in the morning and 3.75 in the afternoon (*p* = 0.044); (ii) sex, with an estimated log_10_ VL of 3.75 in males vs. 3.56 in females (*p* = 0.041); (iii) location, with an estimated log_10_ VL of 3.79 in the in-patients vs. 3.52 in the out-patients (*p* = 0.007) (Table 2). In summary, SARS-CoV-2 VL was significantly higher in samples requested in the afternoon, in males, and in in-patients.

It is noteworthy that out-patients infected with the VIC strain showed an 11-fold increase in VL between morning and afternoon, whereas the Alpha strain only showed a 2.4-fold increase (Figure 1D). We noted significant interactions between sample request time and location (*p* = 0.004), sample request time and SARS-CoV-2 strain (*p* = 0.042), and between age and location (*p* < 0.0001) (Table 2). For example, the largest difference between in-patients and out-patients was seen in the 80–104-year group (estimated Log_10_ VL = 4.08 vs. 3.28) and the smallest difference in the 40–59-year group (estimated Log_10_ VL = 3.69 vs. 3.53). Analysing the data using three time intervals for sample request time (6:00–9:59; 10:00–13:59 and 14:00–17:59) (Table 3), or treating age as a continuous variable and applying a B-spline analysis to account for non-linearity (GLIMMIX method [17]), produced similar results (Table 4).

## 4. Discussion

Our analysis showed a time-of-day influence on SARS-CoV-2 RNA levels in nasal/throat swabs with modest but significantly higher VL estimates in the afternoon (Log_10_ VL 3.7 vs. 3.8 copies/sample, *p* = 0.044) after adjustment for multiple factors. Our observations are consistent with a study from McNaughton et al., who reported a diurnal variation in SARS-CoV-2 PCR test results from >80,000 nasopharyngeal samples, showing a 2-fold variation in test positivity with a peak of positive results at 2 p.m. [18]. In contrast, two studies assessing the time of sampling on SARS-CoV-2 diagnostic test results using repeated saliva collections reported a trend for higher VLs in samples collected earlier in the day [19,20]. However, the conclusions from the latter two studies are limited by the small cohort sizes (n = 16 and n = 13, respectively).

Cortisol levels are known to oscillate in a diurnal manner and a recent study showed this rhythmic pattern was reduced in COVID-19, with a more significant perturbation in hospitalized patients with more severe disease [21]. As circadian amplitude and timing can differ in hospitalized (compared with non-hospitalized) patients, resulting from multiple factors including disease severity, medication and changes in the environment (e.g., lighting) [22,23,24], it is important to compare the diurnal variation in SARS-CoV-2 VL separately between in- and out-patients. We noted a greater difference between the morning and afternoon VL in out-patients infected with VIC (Log_10_ VL 2.68 in AM and 3.74 in PM) compared with Alpha (Log_10_ VL 3.36 in AM and 3.74 in PM), that may relate to different replication rates or sites of infection between the variants [25].

Many respiratory infections follow a seasonal pattern, including COVID-19 [26,27]. Given the link between seasonality and circadian rhythms [28,29], it will be important to identify if SARS-CoV-2 variants evolve and adapt to seasonal effects, as this could influence our timing of booster vaccination programs.

Limitations of this study include the lack of information on actual sample collection time, medical or medication history, dietary information, sleep and shift-work patterns of the participants; and the heterogeneity of the cohort with subjects sampled in different stages of COVID-19—all of which could influence virus replication [30,31]. Our cohort does not include children or clinically vulnerable groups, such as immunocompromised patients. Finally, the clinical relevance of VL measurements and their association with COVID-19 severity is not known [32].

This study highlights the value of recording sample time in clinical and research studies and suggests time-of-day factors should be considered when designing clinical trials to evaluate antiviral drug efficacy where the most frequently measured end point is VL, and when designing epidemiological studies that track viral transmission. These time-of-day-dependent changes in VL could impact the interpretation of results from diagnostic assays, as discussed by McNaughton et al. [18], as well as disease severity and mortality [33]. It will be of interest to analyse the time-of-day dependency of other SARS-CoV-2 variants including Delta and Omicron and to assess the impact of vaccination on VL.

## Figures and Tables

**Table 1:** Number of participants in each sex, age group, in-patient vs. out-patient, and AM/PM category.

	Female				Male				
	In-Patients		Out-Patients		In-Patients		Out-Patients		
Age	AM	PM	AM	PM	AM	PM	AM	PM	Total
18–39	31	39	71	84	23	28	50	77	403
40–59	44	67	56	56	88	84	62	62	519
60–79	90	89	70	66	159	164	67	62	767
80–104	83	87	40	50	56	91	50	46	503
Total	248	282	237	256	326	367	229	247	2192

**Table 2:** Linear mixed-effect modelling results (Type III tests of fixed effects).

Main Effects:	Num DF	F-Value	*p*-Value
Time (AM/PM)	1	4.09	**0.044**
Age	3	0.14	0.935
Sex	1	4.19	**0.041**
In/Out patient	1	7.28	**0.007**
VIC/Alpha	1	2.94	0.087
**Interaction terms:**			
Time × Age	3	1.69	0.169
Time × Sex	1	0.48	0.487
Time × In/Out patient	1	8.45	**0.004**
Time × VIC/Alpha	1	4.17	**0.042**
Age × Sex	3	0.93	0.426
Age × In/Out patient	3	7.93	**<0.0001**
Age × VIC/Alpha	3	2.02	0.11
Sex × In/Out patient	1	2.76	0.097
Sex × VIC/Alpha	1	0.04	0.847
In/Out patient × VIC/Alpha	1	0.18	0.671

Bold values denote statistical significance at the *p* < 0.05 level.

**Table 3:** Analyses with three time groups.

Main Effects:	Num DF	F-Value	*p*-Value
Time (AM/Mid-day/PM)	2	3.09	**0.046**
Age	3	0.44	0.725
Sex	1	1.92	0.166
In/Out patient	1	11.68	**0.0007**
VIC/Alpha	1	4.2	**0.041**
Interaction terms:			
Time × Age	6	1.79	0.099
Time × Sex	2	1.4	0.248
Time × In/Out patient	2	3.17	**0.043**
Time × VIC/Alpha	2	3.8	**0.023**
Age × Sex	3	1.03	0.381
Age × In/Out patient	3	6.98	**0.0001**
Age × VIC/Alpha	3	2.08	0.102
Sex × In/Out patient	1	1.22	0.27
Sex × VIC/Alpha	1	0	0.97
In/Out patient × VIC/Alpha	1	0.48	0.491

Bold values denote statistical significance at the *p* < 0.05 level.

**Table 4:** Analyses with age as a continuous variable.

Main Effects:	Num DF	F-Value	*p*-Value
Time (AM/PM)	1	6.71	**0.0096**
Age (B-spline forms)	6	0.96	0.4482
Sex	1	3.2	0.0737
In/Out patient	1	1.13	0.2882
VIC/Alpha	1	3.64	0.0567
Interaction terms:			
Time × Age	6	2.23	**0.0382**
Time × Sex	1	0.22	0.6354
Time × In/Out patient	1	7.02	**0.0081**
Time × VIC/Alpha	1	4.11	**0.0427**
Age × Sex	6	1.22	0.2949
Age × In/Out patient	6	4.66	**0.0001**
Age × VIC/Alpha	6	1.41	0.2054
Sex × In/Out patient	1	2.81	0.094
Sex × VIC/Alpha	1	0	0.9711
In/Out patient × VIC/Alpha	1	0.45	0.5004

Bold values denote statistical significance at the *p* < 0.05 level.

## Data Availability

The authors declare that all data supporting the findings of this study are available in the article.
